# Enhancing Yeast Surface Display: UPR, ERAD, and ER Dynamics in Recombinant Protein Production

**DOI:** 10.17113/ftb.64.01.26.8764

**Published:** 2026-02-15

**Authors:** Tea Martinić Cezar, Antonia Paić, Bojan Žunar, Igor Stuparević, Vladimir Mrša, Renata Teparić

**Affiliations:** University of Zagreb Faculty of Food Technology and Biotechnology, Pierottijeva 6, Zagreb, Croatia

**Keywords:** yeast surface display, recombinant protein, endoplasmic reticulum-associated degradation (ERAD), unfolded protein response (UPR)

## Abstract

Over the past two decades, the display of various recombinant proteins on the surfaces of microorganisms, particularly yeast, has garnered significant research attention. This method is rapid, simple and cost-effective, combining the biosynthesis and secretion of recombinant proteins with their immobilization on the host cell surface. Proteins synthesized using this technique are transported to the cell surface and incorporated into the cell wall through mild, native processes, avoiding aggressive chemical immobilization methods that often lead to a loss of physiological activity. Surface-displayed proteins are generally more stable and resistant to environmental changes than those in a solution. Depending on the promoter used, cells can continuously renew the recombinant protein on their surface or express it only under certain conditions. Additionally, cells carrying surface-displayed enzymes can be easily separated from the reaction mixture and reused multiple times. These enzymes can also catalyze reactions with substrates that cannot enter the cells, facilitating extracellular synthesis and simplifying product purification. However, the main obstacle to the industrial application of this method is often low efficiency, resulting in limited amounts of displayed protein. The efficiency depends on the processes that the protein undergoes on its way to the cell surface, following the same pathway as native secretory proteins: synthesis in the endoplasmic reticulum (ER), transport to the Golgi, and delivery to the cell surface *via* transport vesicles. Large amounts of secretory proteins can overload the ER, triggering the unfolded protein response (UPR) and endoplasmic reticulum-associated degradation (ERAD). Despite significant improvements for some proteins, a universal system for all recombinant proteins has yet to be developed. However, the complexity of protein processing and secretion pathways suggests that a single system improving productivity for all recombinant proteins is unlikely. Instead, several optimized systems tailored to specific protein structures may be necessary. This article provides an overview of the processes that recombinant proteins intended for surface display undergo on their way to the cell surface in the endoplasmic reticulum and represent a crucial bottleneck for the successful immobilization of recombinant proteins at the cell surface.

## INTRODUCTION

Over the past two decades, the display of various recombinant proteins on the cell surfaces of microorganisms, especially yeast, has been a major focus of research worldwide. This fast, simple and inexpensive method combines the biosynthesis and secretion of recombinant proteins with their immobilization on the surface of the host cell. Different systems for the immobilization of recombinant proteins make it possible to find the optimal solution for each specific case. Recombinant proteins synthesized with this technique are transported to the cell surface and incorporated into the cell wall by mild, native cellular processes. This approach avoids the aggressive chemical immobilization methods that can often lead to loss of physiological activity. In addition, proteins immobilized on the cell surface are generally more stable and resistant to environmental changes than those in a solution. Depending on the promoter used in the constructs, the cells can either continuously renew the recombinant protein on their surface or express it only under certain conditions. This method eliminates the need to isolate, purify and chemically immobilize proteins on a carrier, making the process faster, simpler and less expensive. Another advantage of surface-displayed enzymes is that cells carrying these proteins can be easily separated from the reaction mixture and used multiple times. In addition, surface-displayed enzymes can catalyze reactions with substrates that cannot enter the cells, facilitating extracellular synthesis and simplifying purification of products.

The main obstacle to the industrial application of this method is often the low efficiency, which leads to limited amounts of the displayed protein. The efficiency of surface display depends, among other things, on the processes that the protein undergoes on its way to the cell surface. Recombinant proteins intended for secretion or surface display follow the same pathway as native secretory proteins. They are synthesized in the endoplasmic reticulum (ER), transported to the Golgi and then delivered to the cell surface *via* transport vesicles. In the ER, the proteins are folded and undergo co- and post-translational modifications, including glycosylation. In addition to influencing proper protein maturation, glycosylation of cell wall proteins is required for the assembly of the outer mannan layer, which determines the permeability of the wall (*1*). The degree of protein mannosylation and consequently the thickness and density of the mannan layer could influence the availability of substrates for the enzymes expressed on the cell surface, which in turn affects reaction kinetics and enzyme activity (*2*, *3*).

**Fig. 1:**
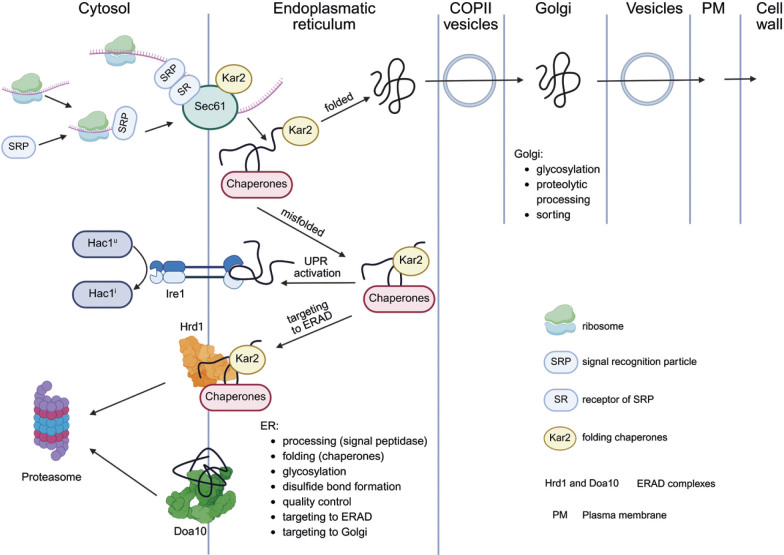
Canonical protein trafficking, unfolded protein response (UPR) and endoplasmic reticulum-associated degradation (ERAD) pathway in yeast cells. Created in BioRender by B. Zunar (2025) https://BioRender.com/s19pm3q

The process of protein folding in the ER is supported and controlled by the action of molecular chaperones. Large amounts of secretory proteins can overload the ER and trigger the unfolded protein response (UPR) and endoplasmic reticulum-associated degradation (ERAD) (Fig. 1). These pathways contribute to reducing ER stress by slowing down overall protein synthesis, increasing the production of chaperones and directing misfolded proteins to the proteasome for degradation. Properly folded and modified proteins are packaged into COPII-coated vesicles and transported to the Golgi for further processing and sorting. Vesicles containing recombinant proteins are transported from the Golgi along the cytoskeleton to the plasma membrane, where they fuse and release their contents into the periplasmic space. The proteins are then bound non-covalently or covalently to the glucan of the cell wall by mechanisms that depend on the surface display model used for their construction. Some proteins are bound to glycosylphosphatidylinositol (GPI) anchors, which are initially incorporated into the plasma membrane and later transferred to the cell wall, where they bind covalently to β-1,6-glucan. Other proteins are covalently bound to the cell wall by ester bonds between specific glutamates in the so-called Pir sequences, which are present in proteins of the PIR family, and β-1,3-glucan. The most common GPI-anchored proteins used for surface display of heterologous proteins are α-agglutinin, a-agglutinin, Cwp2 and Sed1. Yeast a-agglutinin consists of two subunits, one of which (Aga1) is bound to the cell wall *via* a GPI anchor, while the other (Aga2) is connected to Aga1 *via* disulfide bridges. The fusion of the heterologous protein with the C- or N-terminal of the Aga2 subunit results in its immobilization on the cell surface. Finally, some cell wall proteins are just adsorbed non-covalently to cell wall carbohydrates. However, of this group of proteins, only Flo1 is used for cell surface display. Flo1 is a lectin-like protein that contains the flocculation domain near the N-terminus. Numerous cell surface display systems have been constructed in which the N-terminus of the heterologous protein is fused to the C-terminus of the Flo1 flocculation domain. More detailed information on the cell wall proteins used to make recombinant constructs and the strategies used for improvement of their incorporation onto the cell surface can be found in already published review articles (*4*-*6*).

There are indications that the ability of the cell wall to bind covalently embedded proteins is limited by the amount of native cell wall proteins it contains (*7*). *Saccharomyces cerevisiae* has 5 genes encoding Pir proteins and 31 genes encoding GPI-bound proteins, which are thought to be localized in the cell wall, while some of the GPI-bound proteins remain anchored in the membrane and are not transferred to the β-1,6-glucan (*8*, *9*). Thus, the capacity of the cell wall to bind heterologous proteins could be increased by removing autochthonous Pir- or GPI-bound proteins. Finally, mutations in genes encoding proteins involved in endocytosis have been shown to increase the amount of secreted heterologous proteins from yeast cells (*10*), which could also affect the amount of heterologous proteins displayed on the surface.

To improve the industrial applicability of protein surface display, many attempts have been made to optimize various cellular events involved in this process. These include the regulation of protein synthesis, the secretory pathway and endocytosis, modifications of the structure of cell wall carbohydrates and native protein content, and mechanisms for anchoring recombinant proteins in the cell wall. Although significant improvements have been achieved for some proteins, a universal system for the successful immobilization of all recombinant proteins has yet to be developed. This article provides an overview of the processes that recombinant proteins intended for surface display undergo on their way to the cell surface in the endoplasmic reticulum, representing a crucial bottleneck for the successful immobilization of recombinant proteins at the cell surface.

## OVERCOMING THE SURFACE EXPOSURE BARRIER: STRATEGIES FOR STABILIZING PROTEINS IN YEAST DISPLAY

Many proteins are only marginally stable in their native state, and displaying them on the yeast surface can exacerbate folding and stability problems. This phenomenon, termed the ’surface exposure barrier’, refers to the destabilization, misfolding, aggregation or loss of function that can occur when a marginally stable protein is tethered and exposed on the cell surface. Non-native conditions that can create a ’surface exposure barrier’ are an altered environment, loss of native interactions and cofactors, conformational constraints due to binding, (altered) glycosylation and secretory folding and quality control. The extracellular milieu and yeast cell wall environment differ from the cytosol or other native cellular compartments. A protein displayed on the yeast surface is exposed to the surrounding medium, which may have a different pH, redox potential or ionic strength than the protein’s native environment. Optimization of cultivation conditions (pH, temperature, osmolyte addition, *etc.*) can result in subtle differences for certain proteins (*11*). Many proteins rely on partner subunits, ligands or cellular cofactors to fold correctly or remain stable. When a protein is expressed in isolation on the cell surface, these stabilizing interactions may not be present. If the instability of a protein is due to a lack of cofactors or partner subunits, one solution is to provide those partners *in trans*. In yeast display, this might mean co-displaying or co-secreting a binding partner. For example, to display a heterodimeric Fab fragment (which consists of a heavy chain and a light chain), researchers have co-expressed both chains in the same cell: the heavy chain was fused to Aga2 and the light chain was secreted as a soluble protein that associates non-covalently (*12*). In addition, fusion of one terminus of the protein to the anchoring protein constrains that end of the protein, and the tether can perturb the folding or native state stability of the protein. For some enzymes, an N-terminal fusion resulted in an inactive display, while a C-terminal fusion retained activity (*13*). This may be related to how the protein’s own N-terminus is involved in folding or function. A straightforward strategy is therefore to test both N- and C-terminal fusions. The fusion of a protein with an anchor protein can be optimized by adding the linker and adjusting its length. Flexible glycine-serine linkers (*e.g.* GGSGGS repeats) can reduce steric strain and allow the protein domain to fold without colliding with the cell surface or anchor. For example, when displaying enzymes on yeast, a longer linker often enhanced the activity of the enzyme as it could fold into its active conformation without being disrupted by the cell wall (*14*, *15*). N-glycosylation of proteins during secretion can also change the properties of the protein (*15*). Non-native glycosylation may impair folding or function, while the absence of native glycosylation may remove a stabilizing element. One strategy to address this problem is to modify the host glycosylation pathway to better mimic the native context of the protein, which has been shown to improve folding fidelity (*16*). Finally, the proteins displayed on the yeast surface must fold in the oxidizing environment of the endoplasmic reticulum (ER) and pass the cellular quality control points in order to be successfully secreted. A protein that is only marginally stable may misfold during this journey, fail quality control and thus never reach the surface. Indeed, experiments have shown a strong correlation between the thermodynamic stability of a protein and its efficiency of secretion/display in yeast: more stable mutants fold more easily and are displayed in higher copy numbers, while unstable variants are often intercepted by the quality control machinery (*17*). Shusta *et al.* (*18*) found that the ’efficiency of the consecutive kinetic processes of membrane translocation, protein folding, quality control, and vesicular transport’ correlates with protein stability. The secretory pathway thus acts as a filter that excludes marginally stable proteins. This collection of challenges that a protein must overcome to remain correctly folded and functional when displayed on the yeast surface represents an exacerbation of the protein’s marginal stability problem: any tendency to unfold or misfold is amplified by the stress of heterologous secretion and surface binding. As a result, many proteins (especially those that are large, have multiple domains or require delicate interactions) show greatly reduced functional display levels unless measures are taken to stabilize them. More than two decades of methodological refinements and experimental findings have made it clear that stability engineering is not just a side-aspect, but often a central component of yeast display campaigns (*19*). Several strategies have been developed to improve the folding and stability of proteins, enabling their successful display on the yeast surface. One approach is to use prior knowledge of protein structure or evolutionary sequence data to guide stabilizing changes through rational design or consensus design. Rational design might involve the stabilization of a known flexible region (*e.g*. replacing a glycine in a helix with alanine to reduce flexibility, or introducing a salt bridge at a solvent-exposed patch) (*20*). Consensus design is based on the principle that at each position in a protein family, the most frequent amino acid (consensus residue) often contributes to stability, so mutations towards the consensus sequence can stabilize a protein (*15*). A powerful complement to rational design is directed evolution, where randomized libraries of the protein are created and screened for improved stability phenotypes (*19*). In the context of yeast display, several selection pressures can enrich stabilized variants. Shusta *et al.* (*18*) applied a 46 °C heat shock to a scTCR library prior to sorting, effectively selecting mutants based on thermostability. Another approach is to simply sort for high expression levels at normal temperature, following the logic that the yeast’s secretory system itself acts as a selector and that yeast display has an intrinsic link between expression level and stability (*17*). This strategy was used by Traxlmayr *et al.* (*19*) to stabilize already highly thermostable proteins such as the IgG1-Fc domain. They created an error-prone PCR library of an IgG1-Fc and sorted for clones with the highest surface expression and those that retained folding after heat exposure. While glyco-engineering is more common for improving therapeutic protein production, the same principle applies to surface display. Each protein must be evaluated individually, as the effects of glycosylation vary from case to case (*3*, *15*, *16*). Finally, lowering the growth temperature during induction (*e.g*. inducing protein expression at 20 instead of 30 °C) can significantly improve folding yield by slowing down protein synthesis and allowing more time for correct folding (*21*). In addition, the choice of promoter can influence the expression level of protein (*22*). Very strong expression of a difficult protein may overwhelm the folding machinery, whereas a moderately strong promoter can lead to a lower rate of protein synthesis that the cell can handle (resulting in more protein folding rather than aggregating). From a strain engineering perspective, modifying the ER and unfolded protein response (UPR) capacity of the host can be beneficial.

## THE ROLE OF THE STRUCTURE OF THE ENDOPLASMIC RETICULUM IN THE PRODUCTION OF HETEROLOGOUS PROTEINS

The ER is the largest cellular organelle, accounting for about 35 % of the cell volume and extending from the nucleus to the cell membrane as a continuous and complex membrane system organized in sheets and tubules. These morphologically distinct parts of the ER have different functions, with the sheets playing a role in protein maturation and the tubules involved in lipid biosynthesis. The structure of the ER is constantly remodelled and maintained by a series of proteins that regulate its morphology and connect the ER to the microtubules (*23*-*27*). The most important and well-known function of the ER is the synthesis and maturation of secretory proteins. Secretory proteins are synthesized by ribosomes bound to the cytosolic surface of the ER, where the nascent protein chains are translocated into the ER lumen by the Sec61 translocon (*28*). In *S. cerevisiae*, ribosomes are mainly located in the ER sheets (*29*), as the large surface area of the membrane provides sufficient space for ribosome binding and the large volume of its lumen ensures accessibility of the nascent proteins to chaperones required for folding into the native conformation and to enzymes catalyzing post-translational modifications.

The differentiation of the newly synthesized parts of the ER membrane into tubules and sheets is not yet fully understood. It is known that the formation of ER tubules depends on reticulon proteins (*30*). These proteins are incorporated into the cytoplasmic layer of the ER membrane and form ER tubules by oligomerization *via* their specific hydrophobic hairpin structures (*23*, *31*). There are two reticulons (Rtn1 and Rtn2) and one reticulon-like protein (Yop1) in *S. cerevisiae*. Deletion of all three proteins leads to a significant reduction in the amount of ER tubules (*29*), but does not cause defects in vesicular trafficking from the ER and has only a minor negative effect on growth compared to wild-type yeast (*30*). Overexpression of a reticulon protein or alteration of its oligomerization pattern leads to a shift in ER morphology from sheets to tubules (*31*, *32*). Accordingly, expansion of the ER membrane without a parallel increase in reticulon concentration and/or activity leads to the formation of ER sheets. On the other hand, it appears that the sheet structure is stabilized by the Sec61 translocon and the binding of the ribosome (*33*, *34*). The key factor that defines ER morphology in yeast is the proportion between ER surface area and the abundance of Yop1, Rtn1 and Rtn2 (*35*). ER homeostasis is mainly controlled by the unfolded protein response (UPR) signal transduction pathway.

## UPR ACTIVATION DURING ER STRESS

**Fig. 2:**
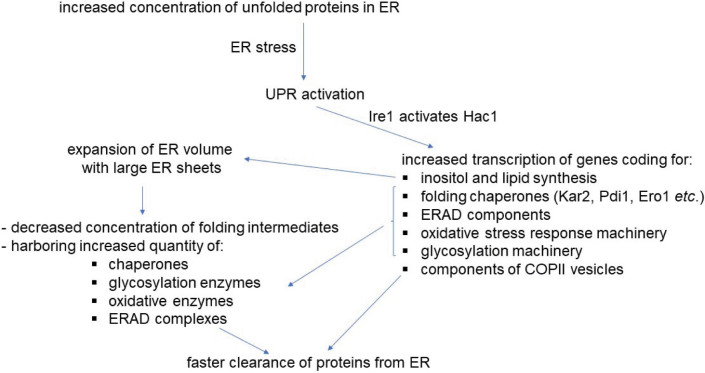
Effects of endoplasmic reticulum (ER) stress and activation of the unfolded protein response (UPR) on the ER structure and activation of ER-associated degradation (ERAD)

When the overexpression of secretory proteins exceeds the folding capacity of the ER, misfolded proteins accumulate in the ER lumen, leading to ER stress, which causes a change in the ER size and shape by activating the UPR (*36*, *37*) (Fig. 2). The expansion of the ER membrane leads to the formation of large ER sheets. Overexpression of the reticulon protein Rtn1 can cause the ER to convert from sheet to tubular shape, but does not alter the effect of increasing ER volume on reducing ER stress (*32*, *38*). Increased ER volume could reduce ER stress by enabling better functioning of membrane-dependent processes (ERAD, glycosylation, *etc*.), harbouring more chaperones, and promoting protein folding by lowering the concentration of folding intermediates that tend to form aggregates through hydrophobic interactions (*39*, *40*). The accumulation of misfolded proteins in the ER activates the UPR sensor protein Ire1, which in turn activates the transcription factor Hac1. Hac1 has been shown to regulate the transcription of approx. 380 genes (*41*). In general, it induces transcription of genes encoding chaperones, which enhances the ER folding capacity, and activates the endoplasmic reticulum-associated degradation (ERAD) pathway (*41*, *42*) to translocate misfolded proteins from the ER to the cytosol, where they are degraded by the proteasome (*43*). The ERAD machinery is constitutively active in the cell, but is additionally activated by Hac1 under ER stress. Mutants in ERAD components constitutively activate the UPR and are hypersensitive to ER stress. The combined deletion of ERAD components and *IRE1* leads to severe synthetic phenotypes (*41*). To increase the processing rate of the secretory pathway and protect the cell from the formation of ROS which occur as a consequence of increased disulfide bond formation during protein maturation, genes involved in response to oxidative stress are also upregulated (*44*), as are genes encoding components of the glycosylation machinery, since glycosylation is important for the proper folding of glycoproteins. In addition, genes encoding components of the post-Golgi, COPI and COPII transport vesicles and enzymes involved in inositol and lipid synthesis are also upregulated to increase ER membrane synthesis and vesicle transport.

The increase in ER volume also depends on the regulation of the transcriptional repressor Opi1, which controls the activity of the heterodimeric transcriptional activator Ino2/Ino4. The deletion of *OPI1* leads to a constitutive activation of the Ino2/Ino4 complex, resulting in increased phospholipid biosynthesis and an expansion of the ER size without increasing the chaperone concentration (*32*). Without Ino2/Ino4 activation, ER expansion is lessened, probably due to reduced biosynthesis of lipids. Moreover, *opi1* mutants and cells with inactive Ino2 exhibit expanded ER sheets independent of Sec61 protein levels, indicating that Sec61 is not limiting for ER sheet formation (*32*). On the other hand, Hac1 activates the Ino2/Ino4 complex, thereby stimulating Ino2/Ino4 activity during ER stress.

Ire1 is a type I transmembrane protein that contains a luminal, a transmembrane and a cytosolic part. The luminal part of Ire1 consists of five subregions (*45*), of which subregions II–IV form a tightly folded so-called core stress sensing region (CSSR). According to crystal structure and systematic mutational analysis, subregion III is a flexible segment, and the Kar2 chaperone binding site is located in subregion V (*46*). Under normal conditions, Ire1 is in a complex with the Kar2 protein. Kar2 is an essential and abundant protein that belongs to the Hsp70 family of chaperones. It supports and controls the folding of secretory proteins in the ER lumen and is involved in the transport of proteins across the ER membrane at the expense of ATP energy (*47*). The synthesis of Kar2 is triggered by the activation of the UPR. Normally, part of Kar2 is bound to immature proteins to support its correct folding, and most of Kar2 is in complex with Ire1. Upon ER stress, the majority of Kar2 is diverted away from Ire1 and bound to misfolded proteins, which is the first step in activating Ire1 (*46*, *48*, *49*). Following the release of Kar2, dimerization of Ire1 occurs *via* the CSSR region, and the CSSR dimer forms a groove similar to the major histocompatibility complex, which is capable of interacting with unfolded proteins (*45*). Further interactions of CSSR with unfolded proteins lead to a change in the conformation of the luminal domain of Ire1, resulting in realignment and activation of the cytosolic domains. In this way, highly oligomerized Ire1 clusters are formed, leading to a fully active Ire1 (*50*). The cytosolic part of Ire 1 contains a protein kinase and an RNase domain. The protein kinase domain triggered by ER stress performs autophosphorylation, followed by activation of the RNase domain, which converts the precursor form of HAC1 mRNA (HAC1u) into the mature form (HAC1i) (*51*, *52*). The HAC1 precursor mRNA (HAC1u) is produced constitutively and contains a specific intron at the 3’ end that is processed only by Ire1 RNase activity (*53*). This intron contains a translation attenuator that forms a loop structure in which the ribosomes are stalled (*54*). The formation of mature mRNA is catalyzed by the splicing activity of Ire1 and the activity of the RNA ligase Rlg1 (*55*). HAC1i is translated into the transcription factor Hac1, which regulates the expression of a number of genes to reduce ER stress (*41*, *56*). The transcription factor Hac1 is a member of the basic leucine zipper (bZIP) family. It forms homodimers and binds to UPRE motifs (UPR element) in promoters of UPR targets (*57*).

After activation, the level of Ire1 RNase activity must be tightly controlled. Chawla *et al*. (*58*) reported that to inactivate the UPR after the restoration of ER function, the kinase domain of Ire1 must recognize and transmit a signal to the RNase domain that ER function has been restored to attenuate the production of Hac1. Their results showed that Ire1 is attenuated by dephosphorylation of the kinase domain and some conformational changes. Restoration of the complex of Ire1 and Kar2, whose transcription is induced by the UPR, also contributes to the attenuation of Ire1 through a negative feedback mechanism. Mutant cells that are unable to attenuate Ire1 activity are less able to survive the extended activation of the UPR, demonstrating the importance of adequate attenuation of the UPR for cell survival (*58*).

## THE ROLE OF ERAD IN THE QUALITY CONTROL OF SECRETORY PROTEINS

Secretory proteins are usually delivered cotranslationally in unfolded form into the lumen of the ER, where molecular chaperones support their folding and keep them in soluble form until folding is complete. Some of the chaperones are part of the ER quality control (ERQC). Correctly folded proteins are then sorted and transported to their final destination, while proteins that fail to fold correctly are targeted for degradation by the ERAD (*59*). Yeast ER chaperones include Kar2 and Lhs1 from the Hsp70 family, the lectin-like Cne1 and the membrane-bound chaperone Rot1, the co-chaperones Scj1 and Jem1, the nucleotide exchange factor Sil1 and the group of thiol oxidoreductases Mpd1, Mpd2, Eps1, Eug1 and Pdi1 (*60*-*65*). The molecular chaperones Kar2, Scj1, Jem1 and Pdi1 bind exposed hydrophobic regions of unfolded proteins (*61*, *65*-*68*).

The method by which ERQC distinguishes between unfolded and folded proteins is not yet fully understood. It is hypothesized that correctly folded proteins form conformational export signals that are recognized by the ERQC sorting mechanism, whereas misfolded proteins are unable to form them and would be retained in the ER. This is supported by the fact that a family of proteins which recognize export signals in mature proteins and concentrate them at ER export sites (so-called ’cargo sorting factors’) is found in the ER (*69*). However, some misfolded proteins possess a functional export signal and can be exported from the ER by COPII vesicles (*70*). This mechanism enables the removal of misfolded proteins from the ER even when the ERAD is saturated (*71*). Some misfolded proteins form insoluble aggregates that are removed by autophagy (*72*).

The best characterized ERAD determinant to date is the modification of the branched glycan chain Glc3-Man9-GlcNAc2 linked to Asn in the protein sequence Asn-X-Ser/Thr (*73*). During the folding process, glucosidase I (Gls1) and glucosidase II (Gls2) sequentially hydrolyze three glucose residues, leaving a truncated Man9-GlcNAc2 chain, which is further truncated by mannosidase I (Mns1) to Man8-GlcNAc2. These reactions are slow, allowing the glycoprotein enough time to fold. If the glycoprotein remains unfolded, it becomes a substrate for the complex of mannosidase Htm1/Mnl1 and protein disulfide isomerase (PDI) (*74*, *75*). The Htm1-PDI complex specifically cleaves the terminal mannose residue in one branch of the glycan chain and exposes an α-1,6-linked mannose, which is the ligand for the Yos9 ERAD receptor (*76*). Inhibition of either of these steps impairs ERAD of glycoproteins (*74*). However, the Man7-GlcNAc2 glycan structure must be bound to a disordered protein segment to signal ERAD (*77*). If folding of the protein is completed in the time required to process the glycan to Man8-GlcNAc2, it escapes Htm1-PDI processing and can leave the ER. It has also been reported that some ERAD substrates are modified by O-mannosylation, although the mechanism by which misfolded proteins are selected for O-mannosylation is not yet understood (*78*, *79*). This is consistent with the fact that the *PMT1* and *PMT2* genes, which encode mannosyltransferases that catalyze O-mannosylation reactions, are targets of the UPR (*41*) and that a number of ER factors, including the Hrd1 complex, are associated with the Pmt1/Pmt2 complex (*80*).

**Fig. 3:**
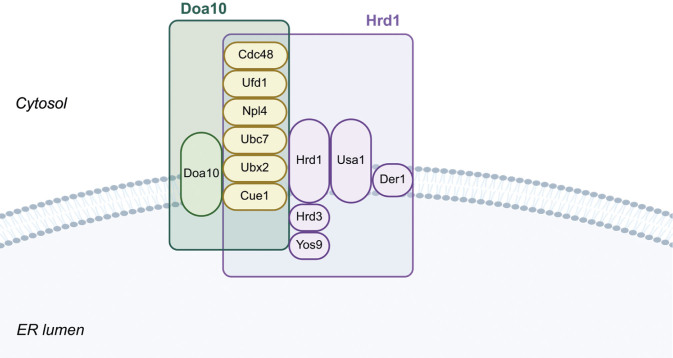
Composition of endoplasmic reticulum-associated degradation (ERAD) complexes. The diagram shows the composition and localization of the distinct components (cytosol, ER membrane, ER lumen) of the individual ERAD complexes. The components of the Doa10 complex are shown in green, the components of the Hrd1 complex in purple and the components that make up both complexes in yellow. Created in BioRender By B. Zunar (2025) https://BioRender.com/s19pm3q

There are two ERAD complexes, Hrd1/Der3 and Doa10, which are located in the ER membrane and recognize, ubiquitinate and translocate misfolded proteins for degradation. These complexes contain the E3 ubiquitin ligase Hrd1 or Doa10 respectively, and a variety of other factors (Fig. 3). Genetic analyses have shown that some of these components, such as Doa10, Hrd1 and Der1, are required for specific substrates, while others (such as Cdc48, Ubc7 and Cue1) are generally required (*81*-*83*). The Cdc48-Npl4-Ufd1-Ubx2 complex and Ubc7 are located in the cytosol and are part of both Doa10 and Hrd1/Der3 ERAD complexes. Both the Doa10 and the Hrd1/Der3 complexes also include the transmembrane component Cue1. Cue1 recruits the Ubc7 E2 ubiquitin-conjugating enzyme to the ERAD complexes. The Hrd1/Der3 complex also contains the membrane component Der1, the luminal components Hrd3 and Yos9 and the cytosolic component Usa1. The transmembrane protein Der1 may be involved in the translocation of misfolded proteins into the cytosol (*84*), while Usa1 acts as a scaffold for the Hrd1/Der3 complex, linking Der1 to Hrd1 (*85*, *86*). The Doa10 complex is specific for membrane proteins with defects in their cytosolic domains (ERAD-C) and Hrd1/Der3 for luminal proteins (ERAD-L) and membrane proteins with defects in their transmembrane segments (ERAD-M) (*81*, *82*, *85*). In ERAD-M mode, the Hrd1 protein itself recognizes defects in transmembrane segments of proteins, whereas in ERAD-L mode, luminal proteins such as Kar2, the Htm1-PDI complex and Yos9 are involved in substrate recognition (*87*, *88*). Kar2 is specific for non-glycosylated and Yos9 for glycosylated substrates, while Hrd3 recognizes unfolded and/or extended segments of polypeptide chains. The Doa10 complex degrades misfolded transmembrane proteins, mostly those with defects in their cytosolic domains, as well as some misfolded cytosolic proteins, and acts as a complement to the Hrd1/Der3 complex (*89*, *90*). There is evidence that cooperation with the cytosolic chaperones Hsp70 Ssa1, Hsp40 Ydj1 and Hsp40 Hlj1 is required for the function of the Doa10 complex (*90*).

The degradation of misfolded proteins takes place in the cytosol, but the mechanism for their translocation from the ER to the cytosol is not yet clear. Moreover, since a wide range of different structural features of potential substrates is possible, there are probably also several mechanisms for retrotranslocation. Polyubiquitination has been shown to be a critical step in the retrotranslocation of both luminal and transmembrane proteins (*90*). Activated ubiquitin is transferred from the cytosolic E1 ubiquitin-activating enzyme Uba1 to the ERAD E2 ubiquitin-conjugating enzymes Ubc6 and Ubc7 and finally to ERAD substrates by the Hrd1/Der3 or Doa10 E3 ubiquitin ligase and the E4 chain-extension enzyme Ufd2 (*90*). The polyubiquitin chains are recognized by the Cdc48 ATPase, which forms a heterotrimeric complex with the cytosolic proteins Ufd1 and Npl4, providing the mechanical force for protein translocation at the expense of ATP hydrolysis (*91*, *92*). However, the components of the translocation channel have not yet been identified, although there are several candidates, including Der1 (*92*) and Hrd1/Der3 (*93*).

Prior to the degradation of glycoproteins by the proteasome, the N-linked glycans must be removed by the cytosolic enzyme Png1 (*94*). The transfer of polyubiquitinated proteins to the proteasome is mediated by the protein Rad23 (*95*). It has been shown that Rad23 can interact with Png1 and Ufd2 and furthermore with Cdc48 *via* Ufd2, potentially linking the ERAD machinery to the proteasome and enabling rapid degradation of ERAD substrates (*96*, *97*).

## MODIFICATIONS OF THE ER, SECRETORY PATHWAY, UPR AND ERAD

Recently, much effort has been devoted to the processes that occur in the ER, *i.e.* cotranslational and posttranslational modifications, folding and transport through the secretory pathway, the quality control system and the mechanisms for the degradation of misfolded proteins (Table 1) (*2*, *3*, *28*, *30*, *31*, *37*, *97*-*118*). It has been shown that the production of recombinant proteins can be improved by genetic modifications targeting the transcriptional level of individual enzymes involved in ER homeostasis or ER membrane expansion processes in general (*40*, *98*). Koskela *et al*. (*98*) showed that overexpression of Ire1, which activates the UPR likely due to a change in the ratio of Ire1 and Kar2, resulted in increased secretion of recombinant proteins. Sheng *et al.* (*100*) showed that overexpression of *IRE1* in a mutant strain lacking Ypt32 (mediates intra-Golgi traffic and the budding of post-Golgi vesicles) increased recombinant protein expression more than twofold. Instead of overexpressing individual chaperone or foldase genes, Valkonen *et al*. (*103*) regulated the expression of *HAC1* and thus affected the entire UPR signalling pathway at once. They showed that deletion of *HAC1* led to decreased production of heterologous proteins, and overexpression of *HAC1* led to increased production of heterologous proteins, suggesting that induction of the UPR favours the production of heterologous proteins, probably by enhancing protein folding and eliminating misfolded proteins by ERAD, which is activated by UPR activation (Fig. 3).

**Table 1:** Effect of modifying the expression of protein components of endoplasmic reticulum (ER), unfolded protein response (UPR), ER-associated degradation (ERAD) and vesicles involved in protein secretion on the ER structure, secretion and surface display of recombinant proteins

Protein	Function/localization	Modification	Effect	Reference
Rtn1 Rtn2 Yop1	reticulon ER proteins	deletion	decrease of tubular ER	(*28*)
Rtn1 Rtn2 Yop1	reticulon ER proteins	overexpression	increase of tubular ER	(*30*, *31*)
Rtn1 Rtn2 Yop1	reticulon ER proteins	deletion	increased volume of ER, attenuated UPR	(*37*)
Opi1	transcription regulator	deletion	increased volume of ER, attenuated UPR	(*37*)
Ire1	UPR sensor protein in ER	overexpression	increased secretion of recombinant proteins	(*97*-*99*)
Hac1	transcription regulator	deletion	decreased production of recombinant proteins	(*100*)
Hac1	transcription regulator	overexpression	increased production of recombinant proteins	(*31*, *100*-*102*)
Pdi1	ER chaperon	overexpression	increased secretion of recombinant proteins	(*103*-*105*)
Kar2	ER chaperon	overexpression	increased secretion of recombinant proteins	(*104*, *106*, *107*)
Ubc7	ERAD component	deletion	accumulation of misfolded proteins in the ER	(*108*-*110*)
Hrd1	ERAD component	deletion	accumulation of misfolded proteins in the ER, decreased production of recombinant proteins	(*108*-*111*)
Hrd3	ERAD component	deletion	decreased production of recombinant proteins	(*111*)
Ubc7	ERAD component	deletion	decreased production of recombinant proteins	(*111*)
Yos9	ERAD component	deletion	decreased production of recombinant proteins	(*111*)
Htm1	mannosidase in ER	deletion	increased production of recombinant proteins	(*111*)
Sec12 Sec13 Erv25 Bos1	sorting proteins in ER to Golgi transport vesicles	overexpression	increased secretion of CelA endoglucanase	(*112*)
Sso1 Snc2 Sec1 Sec9	sorting proteins in Golgi to plasma membrane transport vesicles	overexpression	increased secretion of β-glucosidase BGL1	(*112*)
Sec16	sorting proteins in ER to Golgi transport vesicles	overexpression	increased secretion of recombinant proteins	(*113*)
Cog5	sorting proteins in ER to Golgi transport vesicles	deletion	decreased α-amylase secretion	(*114*)
Erv29	sorting proteins in ER to Golgi transport vesicles	deletion	decreased α-amylase secretion	(*115*)
Gos1	retrograde Golgi transport vesicle	deletion	increased α-amylase secretion	(*114*)
Vps5	transport Golgi to endosome vesicle	deletion	enhanced secretion of recombinant protein	(*115*)
Vps17	transport Golgi to endosome vesicle	deletion	enhanced secretion of recombinant protein	(*115*)
Mnn2 Mnn11	protein mannosylation/Golgi	deletion	increased production of recombinant cellulases	(*2*, *116*)
Och1	α-1,6-mannosyltransferase/Golgi	deletion	increased production of human tissue-type plasminogen activator	(*3*, *117*)
Mnn10	protein mannosylation/Golgi	deletion	improved secretion of recombinant proteins and invertase	(*118*)
Mnn1 Mnn9	protein mannosylation/Golgi	deletion	improved production of recombinant proteins	(*3*)

Robinson *et al*. (*106*) showed that overexpression of *PDI* led to increased secretion of human growth factor B and *Schizosaccharomyces pombe* acid phosphatase. Overproduction of Kar2 increased the secretion of bovine prochymosin (*104*), and co-expression of Kar2 and Pdi increased the secretion of single-chain antibody fragments (*116*).

An additional step to increase recombinant protein production was shown to be the regulation of N-glycosylation. Disruption of the genes coding for Mnn2, Mnn10, Mnn11 and Och1 improved the production of different recombinant proteins (*2*, *108*, *117*-*119*). Tang *et al.* (*3*) investigated the effect of N-glycosylation modification on the secretion of three recombinant cellulases, Cel3A (*Saccharomycopsis fibuligera* β-glucosidase), CelA (*Clostridium thermocellum* endoglucanase) and Cel7A (*Trichoderma reesei* cellobiohydrolase I) that were N-hyperglycosylated when expressed in *S. cerevisiae*. In that work, strains with deletions in *OCH1*, *MNN1* and *MNN9* (crucial Golgi mannosyltransferase genes) were used in order to block the hypermannosylation. Results showed a significant increase in extracellular cellulase activities, that was primarily caused by increased protein production. Authors also noticed that the improvement in protein production might be a result of the up-regulation of main components in the secretory pathway, as well as of the damaged cell wall integrity. Namely, genes *SSA1* and *KAR2* (protein folding-related), *SNC2*, *BOS1*, *SSO1* and *ERV25* (protein trafficking-related), and *DER1* and *HRD3* (ERAD-related), were up-regulated in constructed strains.

Studies have been conducted on the effects of a single deletion of ERAD components alone or the deletion of individual ERAD components in combination with a deletion of *IRE1*. It has been found that a single deletion of *UBC7* or *HRD1* leads to slower degradation of ERAD substrates and an accumulation of misfolded substrates in the ER (*109*-*111*). Single deletions of *HRD1*, *HRD3* and *UBC7* showed a slight decrease in the production of recombinant protein, while a more pronounced decrease was observed after deletion of *YOS9* (*105*). However, a slight increase in production was observed after the deletion of the gene *HTM1* (*105*), which encodes the mannosidase responsible for the exposure of an α-1,6-linked mannose required for the efficient binding of the ERAD-targeted glycoprotein to Yos9 (*74*). This could be due to the fact that the *Δhtm1* strain takes longer to fold and secrete the glycoprotein. While deletion of *IRE1* in wild-type cells greatly reduced the production of recombinant proteins, deletion of *IRE1* in the *Δhtm1* and *Δyos9* strains slightly reversed the effects observed in these single mutants. In addition, the *Δyos9Δire1* and *Δhtm1Δire1* strains showed a longer retention of the recombinant proteins in the ER and their slower secretion from the cells (*105*). Similarly, the expression of a number of recombinant proteins in *Pichia pastoris* was improved by coexpression of the Kar2 or Pdi1 (*101*, *107*), Hac1 (*99*, *102*), Ire1 (*120*) and some other co-chaperones (*101*, *120*, *121*) as nicely reviewed in the recent paper by Raschmanová *et al.* (*112*).

A seminal study by Tang *et al.* (*113*) investigated the engineering of vesicle trafficking in *S. cerevisiae* to improve both the extracellular activity and surface display efficiency of cellulases. In this work, components such as Sec12, Sec13, Erv25 and Bos1 were overexpressed to enhance protein transport from the ER to the Golgi, ultimately leading to improved secretion of *Clostridium thermocellum* endoglucanase (CelA). The study further revealed that engineering components in the Golgi-to-plasma membrane trafficking pathway, such as SNARE proteins including Sso1, Snc2, Sec1 and Sec9, had a protein-specific impact; certain cellulases, for instance, experienced enhanced secretion when these genes were upregulated. An important aspect of these modifications is the differential effect that engineering the vesicle trafficking system has on various proteins. While CelA secretion predominantly benefits from modifications in early vesicle transport events (ER to Golgi), the efficient secretion of cellulases like β-glucosidase (BGL1) relies more on enhancements in the later stages (Golgi to plasma membrane). This specificity indicates that distinct proteins possess unique limitations in protein transport, and therefore, the optimization strategy must be tailored to the particular heterologous protein of interest. At the same time display efficiency of CelA and BGL1 fused with a-agglutinin was improved in these mutants. Such results indicate that engineering the vesicle trafficking pathway is important step for regulating both recombinant protein secretion and display.

Bao *et al.* (*114*) showed that the moderate expression of *SEC16* decreases ER stress by increasing COPII formation and the number of ER exit sites, enhancing protein secretion. This was shown to be good general strategy to increase the secretion of a number of recombinant proteins including *Trichoderma reesei* endoglucanase I and *Rhizopus oryzae* glucan-1,4-α-glucosidase. Huang *et al.* (*115*), using microfluidic screening and whole-genome sequencing, identified several genes involved in the secretory and trafficking pathways whose mutations significantly affected the secretion capacity of the mutant cells. The deletion of genes coding for proteins associated with COPII vesicles, such as *COG5* and *ERV29*, decreased α-amylase secretion, while the deletion of *GOS1*, involved in the retrograde Golgi traffic, increased the secretion efficiency. Furthermore, the deletion of genes *VPS5* and *VPS17*, coding for proteins important for transport between the Golgi and endosome, significantly enhanced the secretion of recombinant protein (*122*).

Besada-Lombana and Da Silva (*123*) combined multiple deletions that simultaneously affected multiple points in the secretory pathway. They enhanced the cotranslational translocation of protein into the ER by fusing the signal peptide of the oligosaccharyltransferase Ost1 α subunit to the pro-region of the MFα1 leader sequence. Then, they expanded the ER through *PAH1* deletion, overexpressed *ERV29* (an ER transmembrane receptor needed for protein packing into COPII vesicles) and limited ERAD activity *via* deletion of *DER1*. Expression of a fungal β-glucosidase, a bacterial endoglucanase and a single-chain antibody fragment in this engineered strain resulted in different results for each protein, ranging from 5.8- to 11-fold increase compared to the wt strain.

## CONCLUSIONS

Over the past decade, numerous attempts have been made to modify *Saccharomyces cerevisiae* to improve the production of recombinant proteins. These included the improvement of vector systems, promoters and the signal sequences for secretion, interventions in folding and post-translational modifications as well as the optimization of growth conditions and fermentation. However, none of these attempts has led to a host strain and/or a process that could be successfully used for all recombinant proteins. This is due to the enormous complexity of protein processing and the secretion pathway as well as the great variability of heterologous protein structures. One of the most critical steps in the synthesis of recombinant proteins destined for surface display occurs in the endoplasmic reticulum (ER), where recombinant proteins accumulate in high concentrations due to the high production of these proteins, which is usually achieved by using strong promoters upstream of the structural genes that encode them. This phenomenon burdens the secretory pathway and causes ER stress, leading to the activation of the unfolded protein response (UPR) and endoplasmic reticulum-associated degradation (ERAD) pathways. Different, sometimes even contradictory, results obtained from the inactivation or overexpression of certain proteins in the UPR and ERAD pathways indicate the close link between these two processes and the need for their coordination and balance. The results in the literature show that for improved productivity in the production of some recombinant proteins, it is necessary to accelerate the UPR and/or ERAD to speed up the secretion of the synthesized proteins and increase the concentration of chaperones and other enzymes required for the processes of folding and post-translational modifications. However, for other types of recombinant proteins, the productivity of their synthesis has been shown to be positively affected by slowing down the UPR and/or ERAD, giving newly synthesized proteins more time to adopt their final conformation and preventing their too rapid recruitment for degradation in proteasomes. It can therefore be assumed that it will probably not be possible to create a single, universal system that would improve cell productivity for all types of recombinant proteins. Instead, several different systems with optimized conditions for the synthesis of specific recombinant proteins that share some common structural features should be established.

## References

[1] Orlean P. Architecture and biosynthesis of the *Saccharomyces cerevisiae* cell wall. Genetics. 2012;192(3):775–818. 10.1534/genetics.112.144485 23135325 PMC3522159

[2] Matsuoka H Hashimoto K Saijo A Takada Y Kondo A Ueda M Cell wall structure suitable for surface display of proteins in *Saccharomyces cerevisiae.* Yeast. 2014;31(2):67–76. 10.1002/yea.2995 24357429

[3] Tang H Wang S Wang J Song M Xu M Zhang M N-hypermannose glycosylation disruption enhances recombinant protein production by regulating secretory pathway and cell wall integrity in *Saccharomyces cerevisiae.* Sci Rep. 2016;6:25654. 10.1038/srep25654 27156860 PMC4860636

[4] Lozančić M. Hossain ASk, Mrša V, Teparić R. Surface display - an alternative to classic enzyme immobilization. Catalysts. 2019;9(9):728. 10.3390/catal9090728

[5] Zhang C Chen H Zhu Y Zhang Y Li X Wang F. *Saccharomyces cerevisiae* cell surface display technology: Strategies for improvement and applications. Front Bioeng Biotechnol. 2022;10:1056804. 10.3389/fbioe.2022.1056804 36568309 PMC9767963

[6] Teymennet-Ramírez KV Martínez-Morales F Trejo-Hernández MR. Yeast surface display system: Strategies for improvement and biotechnological applications. Front Bioeng Biotechnol. 2022;9:794742. 10.3389/fbioe.2021.794742 35083204 PMC8784408

[7] Hossain AS Teparić R Mrša V. Comparison of two models of surface display of xylose reductase in the *Saccharomyces cerevisiae* cell wall. Enzyme Microb Technol. 2019;123:8–14. 10.1016/j.enzmictec.2019.01.005 30686349

[8] Yin QY de Groot PW Dekker HL de Jong L Klis FM de Koster CG. Comprehensive proteomic analysis of *Saccharomyces cerevisiae* cell walls: Identification of proteins covalently attached *via* glycosylphosphatidylinositol remnants or mild alkali-sensitive linkages. J Biol Chem. 2005;280(21):20894–901. 10.1074/jbc.M500334200 15781460

[9] Lozančić M Žunar B Hrestak D Lopandić K Teparić R Mrša V. Systematic comparison of cell wall-related proteins of different yeasts. J Fungi (Basel). 2021;7(2):128. 10.3390/jof7020128 33572482 PMC7916363

[10] Rodríguez-Limas WA Tannenbaum V Tyo KEJ. Blocking endocytotic mechanisms to improve heterologous protein titers in *Saccharomyces cerevisiae.* Biotechnol Bioeng. 2015;112(2):376–85. 10.1002/bit.25360 25154809

[11] Tanino T Noguchi E Kimura S Sahara H Hata Y Fukuda H Effect of cultivation conditions on cell-surface display of Flo1 fusion protein using sake yeast. Biochem Eng J. 2007;33(3):232–7. 10.1016/j.bej.2006.11.001

[12] van den Beucken T Pieters H Steukers M van der Vaart M Ladner RC Hoogenboom HR Affinity maturation of Fab antibody fragments by fluorescent-activated cell sorting of yeast - displayed libraries. FEBS Lett. 2003;546(2-3):288–94. 10.1016/S0014-5793(03)00602-1 12832056

[13] Wang Z Mathias A Stavrou S Neville DM. A new yeast display vector permitting free scFv amino termini can augment ligand binding affinities. Protein Eng Des Sel. 2005;18(7):337–43. 10.1093/protein/gzi036 15976011

[14] Lown PS Cai JJ Ritter SC Otolski JJ Wong R Hackel BJ. Extended yeast surface display linkers enhance the enrichment of ligands in direct mammalian cell selections. Protein Eng Des Sel. 2021;34:gzab004. 10.1093/protein/gzab004 33880560 PMC8058008

[15] Zahradník J Dey D Marciano S Kolářová L Charendoff CI Subtil A, Schreiber G A protein-engineered, enhanced yeast display platform for rapid evolution of challenging targets. ACS Synth Biol. 2021;10(12):3445–60. 10.1021/acssynbio.1c00395 34809429 PMC8689690

[16] Xu L Bai X Oh EJ. Strategic approaches for designing yeast strains as protein secretion and display platforms. Crit Rev Biotechnol. 2025;45(3):491–508. 10.1080/07388551.2024.2385996 39138023

[17] Könning D Kolmar H. Beyond antibody engineering: Directed evolution of alternative binding scaffolds and enzymes using yeast surface display. Microb Cell Fact. 2018;17:32. 10.1186/s12934-018-0881-3 29482656 PMC6389260

[18] Shusta EV Holler PD Kieke MC Kranz DM Wittrup KD. Directed evolution of a stable scaffold for T-cell receptor engineering. Nat Biotechnol. 2000;18:754–9. 10.1038/77325 10888844

[19] Traxlmayr MW Obinger C. Directed evolution of proteins for increased stability and expression using yeast display. Arch Biochem Biophys. 2012;526(2):174–80. 10.1016/j.abb.2012.04.022 22575387

[20] Van Blarcom T, Rossi A, Foletti D, Sundar P, Pitts S, Melton Z, *et al*. Epitope mapping using yeast display and next generation sequencing. In: Rockberg J, Nilvebrant J, editors. Epitope mapping protocols. Methods in molecular biology, vol. 1785. New York, NY, USA: Humana Press; 2018. pp. 89-118. 10.1007/978-1-4939-7841-0_7 29714014

[21] Hackel BJ Huang D Bubolz JC Wang XX Shusta EV. Production of soluble and active transferrin receptor-targeting single-chain antibody using *Saccharomyces cerevisiae.* Pharm Res. 2006;23(4):790–7. 10.1007/s11095-006-9778-7 16550469

[22] Feng X Marchisio MA. *Saccharomyces cerevisiae* promoter engineering before and during the synthetic biology era. Biology (Basel). 2021;10(6):504. 10.3390/biology10060504 34204069 PMC8229000

[23] Hu J Shibata Y Voss C Shemesh T Li Z Coughlin M Membrane proteins of the endoplasmic reticulum induce high-curvature tubules. Science. 2008;319(5867):1247–50. 10.1126/science.1153634 18309084

[24] Wang S Tukachinsky H Romano FB Rapoport TA. Cooperation of the ER-shaping proteins atlastin, lunapark, and reticulons to generate a tubular membrane network. eLife. 2016;5:e18605. 10.7554/eLife.18605 27619977 PMC5021524

[25] Shibata Y Shemesh T Prinz WA Palazzo AF Kozlov MM Rapoport TA. Mechanisms determining the morphology of the peripheral ER. Cell. 2010;143(5):774–88. 10.1016/j.cell.2010.11.007 21111237 PMC3008339

[26] Lin S Sun S Hu J. Molecular basis for sculpting the endoplasmic reticulum membrane. Int J Biochem Cell Biol. 2012;44(9):1436–43. 10.1016/j.biocel.2012.05.013 22640864

[27] Ogawa-Goto K Tanaka K Ueno T Tanaka K Kurata T Sata T p180 is involved in the interaction between the endoplasmic reticulum and microtubules through a novel microtubule-binding and bundling domain. Mol Biol Cell. 2007;18(10):3741–51. 10.1091/mbc.e06-12-1125 17634287 PMC1995732

[28] Sicking M Lang S Bochen F Roos A Drenth JPH Zakaria M Complexity and specificity of Sec61-channelopathies: Human diseases affecting gating of the Sec61 complex. Cells. 2021;10:1036. 10.3390/cells10051036 33925740 PMC8147068

[29] West M Zurek N Hoenger A Voeltz GK. A 3D analysis of yeast ER structure reveals how ER domains are organized by membrane curvature. J Cell Biol. 2011;193:333–46. 10.1083/jcb.201011039 21502358 PMC3080256

[30] Voeltz GK Prinz WA Shibata Y Rist JM Rapoport TA. A class of membrane proteins shaping the tubular endoplasmic reticulum. Cell. 2006;124:573–86. 10.1016/j.cell.2005.11.047 16469703

[31] Shibata Y Voss C Rist JM Hu J Rapoport TA Prinz WA The reticulon and DP1/Yop1p proteins form immobile oligomers in the tubular endoplasmic reticulum. J Biol Chem. 2008;283:18892–904. 10.1074/jbc.M800986200 18442980 PMC2441541

[32] Schuck S Prinz WA Thorn KS Voss C Walter P. Membrane expansion alleviates endoplasmic reticulum stress independently of the unfolded protein response. J Cell Biol. 2009;187:525–36. 10.1083/jcb.200907074 19948500 PMC2779237

[33] Shibata Y Voeltz GK Rapoport TA. Rough sheets and smooth tubules. Cell. 2006;126:435–9. 10.1016/j.cell.2006.07.019 16901774

[34] Puhka M Vihinen H Joensuu M Jokitalo E. Endoplasmic reticulum remains continuous and undergoes sheet-to-tubule transformation during cell division in mammalian cells. J Cell Biol. 2007;179:895–909. 10.1083/jcb.200705112 18056408 PMC2099207

[35] Westrate LM Lee JE Prinz WA Voeltz GK. Form follows function: the importance of endoplasmic reticulum shape. Annu Rev Biochem. 2015;84:791–811. 10.1146/annurev-biochem-072711-163501 25580528

[36] Federovitch CM Ron D Hampton RY. The dynamic ER: experimental approaches and current questions. Curr Opin Cell Biol. 2005;17:409–14. 10.1016/j.ceb.2005.06.010 15975777

[37] Borgese N Francolini M Snapp E. Endoplasmic reticulum architecture: structures in flux. Curr Opin Cell Biol. 2006;18:358–64. 10.1016/j.ceb.2006.06.008 16806883 PMC4264046

[38] Niemelä LRK Koskela EV Frey AD. Modification of the endoplasmic reticulum morphology enables improved recombinant antibody expression in *Saccharomyces cerevisiae.* J Biotechnol. 2024;387:1–11. 10.1016/j.jbiotec.2024.03.009 38555020

[39] Apetri AC Horwich AL. Chaperonin chamber accelerates protein folding through passive action of preventing aggregation. Proc Natl Acad Sci USA. 2008;105:17351–5. 10.1073/pnas.0809794105 18987317 PMC2579888

[40] de Ruijter JC Koskela EV Frey AD. Enhancing antibody folding and secretion by tailoring the *Saccharomyces cerevisiae* endoplasmic reticulum. Microb Cell Fact. 2016;15:87. 10.1186/s12934-016-0488-5 27216259 PMC4878073

[41] Travers KJ Patil CK Wodicka L Lockhart DJ Weissman JS Walter P. Functional and genomic analyses reveal an essential coordination between the unfolded protein response and ER-associated degradation. Cell. 2000;101:249–58. 10.1016/S0092-8674(00)80835-1 10847680

[42] Cox JS Chapman RE Walter P. The unfolded protein response coordinates the production of endoplasmic reticulum protein and endoplasmic reticulum membrane. Mol Biol Cell. 1997;8:1805–14. 10.1091/mbc.8.9.1805 9307975 PMC305738

[43] Vembar SS Brodsky JL. One step at a time: endoplasmic reticulum associated degradation. Nat Rev Mol Cell Biol. 2008;9:944–57. 10.1038/nrm2546 19002207 PMC2654601

[44] Haynes CM Titus EA Cooper AA. Degradation of misfolded proteins prevents ER-derived oxidative stress and cell death. Mol Cell. 2004;15:767–76. 10.1016/j.molcel.2004.08.025 15350220

[45] Credle JJ Finer-Moore JS Papa FR Stroud RM Walter P. On the mechanism of sensing unfolded protein in the endoplasmic reticulum. Proc Natl Acad Sci USA. 2005;102:18773–84. 10.1073/pnas.0509487102 16365312 PMC1316886

[46] Kimata Y Oikawa D Shimizu Y Ishiwata-Kimata Y Kohno K. A role for BiP as an adjustor for the endoplasmic reticulum stress-sensing protein Ire1. J Cell Biol. 2004;167:445–56. 10.1083/jcb.200405153 15520230 PMC2172501

[47] Hernández-Elvira M Torres-Quiroz F Escamilla-Ayala A Domínguez-Martin E Escalante R Kawasaki L The unfolded protein response pathway in the yeast *Kluyveromyces lactis*. A comparative view among yeast species. Cells. 2018;7(8):106. 10.3390/cells7080106 30110882 PMC6116095

[48] Bertolotti A Zhang Y Hendershot LM Harding HP Ron D. Dynamic interaction of BiP and ER stress transducers in the unfolded protein response. Nat Cell Biol. 2000;2:326–32. 10.1038/35014014 10854322

[49] Okamura K Kimata Y Higashio H Tsuru A Kohno K. Dissociation of Kar2p/BiP from an ER sensory molecule, Ire1p, triggers the unfolded protein response in yeast. Biochem Biophys Res Commun. 2000;279:445–50. 10.1006/bbrc.2000.3987 11118306

[50] Kimata Y Ishiwata-Kimata Y Ito T Hirata A Suzuki T Oikawa D Two regulatory steps of ER-stress sensor Ire1 involving its cluster formation and interaction with unfolded proteins. J Cell Biol. 2007;179:75–86. 10.1083/jcb.200704166 17923530 PMC2064738

[51] Papa FR Zhang C Shokat K Walter P. Bypassing a kinase activity with an ATP-competitive drug. Science. 2003;302:1533–7. 10.1126/science.1090031 14564015

[52] Cox JS Walter P. A novel mechanism for regulating activity of a transcription factor that controls the unfolded protein response. Cell. 1996;87:391–404. 10.1016/S0092-8674(00)81360-4 8898193

[53] Sidrauski C Walter P. The transmembrane kinase Ire1p is a site-specific endonuclease that initiates mRNA splicing in the unfolded protein response. Cell. 1997;90:1031–9. 10.1016/S0092-8674(00)80369-4 9323131

[54] Aragón T Van Anken E Pincus D Serafimova IM Korennykh AV Rubio CA Messenger RNA targeting to endoplasmic reticulum stress signalling sites. Nature. 2009;457:736–40. 10.1038/nature07641 19079237 PMC2768538

[55] van Anken E Pincus D Coyle S Aragón T Osman C Lari F Specificity in endoplasmic reticulum-stress signaling in yeast entails a step-wise engagement of HAC1 mRNA to clusters of the stress sensor Ire1. eLife. 2014;3:e05031. 10.7554/eLife.05031 25549299 PMC4279078

[56] Kimata Y Ishiwata-Kimata Y Yamada S Kohno K. Yeast unfolded protein response pathway regulates expression of genes for anti-oxidative stress and for cell surface proteins. Genes Cells. 2006;11:59–69. 10.1111/j.1365-2443.2005.00921.x 16371132

[57] Fordyce PM Pincus D Kimmig P Nelson CS El-Samad H Walter P Basic leucine zipper transcription factor Hac1 binds DNA in two distinct modes as revealed by microfluidic analyses. Proc Natl Acad Sci USA. 2012;109:E3084–93. 10.1073/pnas.1212457109 23054834 PMC3494901

[58] Chawla A Chakrabarti S Ghosh G Niwa M. Attenuation of yeast UPR is essential for survival and is mediated by IRE1 kinase. J Cell Biol. 2011;193:41–50. 10.1083/jcb.201008071 21444691 PMC3082189

[59] Thibault G Ng DTW. The endoplasmic reticulum-associated degradation pathways of budding yeast. Cold Spring Harb Perspect Biol. 2012;4:a013193. 10.1101/cshperspect.a013193 23209158 PMC3504435

[60] Tyson JR Stirling CJ. *LHS1* and *SIL1* provide a luminal function that is essential for protein translocation into the endoplasmic reticulum. EMBO J. 2000;19:6440–52. 10.1093/emboj/19.23.6440 11101517 PMC305876

[61] Nishikawa SI Fewell SW Kato Y Brodsky JL Endo T. Molecular chaperones in the yeast endoplasmic reticulum maintain the solubility of proteins for retrotranslocation and degradation. J Cell Biol. 2001;153:1061–70. 10.1083/jcb.153.5.1061 11381090 PMC2174341

[62] Zhang Y Nijbroek G Sullivan ML McCracken AA Watkins SC Michaelis S Hsp70 molecular chaperone facilitates endoplasmic reticulum-associated protein degradation of cystic fibrosis transmembrane conductance regulator in yeast. Mol Biol Cell. 2001;12:1303–14. 10.1091/mbc.12.5.1303 11359923 PMC34585

[63] Wang Q Chang A. Substrate recognition in ER-associated degradation mediated by Eps1, a member of the protein disulfide isomerase family. EMBO J. 2003;22:3792–802. 10.1093/emboj/cdg378 12881414 PMC169051

[64] Takeuchi M Kimata Y Kohno K. *Saccharomyces cerevisiae* Rot1 is an essential molecular chaperone in the endoplasmic reticulum. Mol Biol Cell. 2008;19:3514–25. 10.1091/mbc.e07-12-1289 18508919 PMC2488298

[65] Grubb S Guo L Fisher EA Brodsky JL. Protein disulfide isomerases contribute differentially to the endoplasmic reticulum-associated degradation of apolipoprotein B and other substrates. Mol Biol Cell. 2012;23:520–32. 10.1091/mbc.e11-08-0704 22190736 PMC3279382

[66] Silberstein S Schlenstedt G Silver PA Gilmore R. A role for the DnaJ homologue Scj1p in protein folding in the yeast endoplasmic reticulum. J Cell Biol. 1998;143:921–33. 10.1083/jcb.143.4.921 9817751 PMC2132949

[67] Gillece P Luz JM Lennarz WJ de La Cruz FJ Romisch K. Export of a cysteine-free misfolded secretory protein from the endoplasmic reticulum for degradation requires interaction with protein disulfide isomerase. J Cell Biol. 1999;147:1443–56. 10.1083/jcb.147.7.1443 10613903 PMC2174254

[68] Thibault G Ismail N Ng DT. The unfolded protein response supports cellular robustness as a broad-spectrum compensatory pathway. Proc Natl Acad Sci USA. 2011;108:20597–602. 10.1073/pnas.1117184109 22143797 PMC3251055

[69] Dancourt J Barlowe C. Protein sorting receptors in the early secretory pathway. Annu Rev Biochem. 2010;79:777–802. 10.1146/annurev-biochem-061608-091319 20533886

[70] Kawaguchi S Hsu CL Ng DT. Interplay of substrate retention and export signals in endoplasmic reticulum quality control. PLoS ONE. 2010;5:e15532. 10.1371/journal.pone.0015532 21151492 PMC2991357

[71] Spear ED Ng DT. Stress tolerance of misfolded carboxypeptidase Y requires maintenance of protein trafficking and degradative pathways. Mol Biol Cell. 2003;14:2756–67. 10.1091/mbc.e02-11-0717 12857862 PMC165674

[72] Ishida Y Nagata K. Autophagy eliminates a specific species of misfolded procollagen and plays a protective role in cell survival against ER stress. Autophagy. 2009;5:1217–9. 10.4161/auto.5.8.10168 19829057

[73] Knop M Hauser N Wolf DH. N-Glycosylation affects endoplasmic reticulum degradation of a mutated derivative of carboxypeptidase yscY in yeast. Yeast. 1996;12:1229–38. 10.1002/(SICI)1097-0061(19960930)12:12<1229::AID-YEA15>3.0.CO;2-H 8905927

[74] Clerc S Hirsch C Oggier DM Deprez P Jakob C Sommer T Htm1 protein generates the N-glycan signal for glycoprotein degradation in the endoplasmic reticulum. J Cell Biol. 2009;184:159–72. 10.1083/jcb.200809198 19124653 PMC2615083

[75] Gauss R Kanehara K Carvalho P Ng DT Aebi M. A complex of Pdi1p and the mannosidase Htm1p initiates clearance of unfolded glycoproteins from the endoplasmic reticulum. Mol Cell. 2011;42:782–93. 10.1016/j.molcel.2011.04.027 21700223

[76] Quan EM Kamiya Y Kamiya D Denic V Weibezahn J Kato K Defining the glycan destruction signal for endoplasmic reticulum-associated degradation. Mol Cell. 2008;32:870–7. 10.1016/j.molcel.2008.11.017 19111666 PMC2873636

[77] Xie W Kanehara K Sayeed A Ng DT. Intrinsic conformational determinants signal protein misfolding to the Hrd1/Htm1 endoplasmic reticulum-associated degradation system. Mol Biol Cell. 2009;20:3317–29. 10.1091/mbc.e09-03-0231 19458187 PMC2710827

[78] Nakatsukasa K Okada S Umebayashi K Fukuda R Nishikawa S Endo T. Roles of O-mannosylation of aberrant proteins in reduction of the load for endoplasmic reticulum chaperones in yeast. J Biol Chem. 2004;279:49762–72. 10.1074/jbc.M403234200 15377669

[79] Hirayama H Fujita M Yoko-o T Jigami Y. O-mannosylation is required for degradation of the endoplasmic reticulum-associated degradation substrate Gas1p *via* the ubiquitin/proteasome pathway in *Saccharomyces cerevisiae.* J Biochem. 2008;143:555–67. 10.1093/jb/mvm249 18182384

[80] Goder V Melero A. Protein O-mannosyltransferases participate in ER protein quality control. J Cell Sci. 2011;124:144–53. 10.1242/jcs.072181 21147851

[81] Taxis C Hitt R Park SH Deak PM Kostova Z Wolf DH. Use of modular substrates demonstrates mechanistic diversity and reveals differences in chaperone requirement of ERAD. J Biol Chem. 2003;278:35903–13. 10.1074/jbc.M301080200 12847107

[82] Huyer G Piluek WF Fansler Z Kreft SG Hochstrasser M Brodsky JL Distinct machinery is required in *Saccharomyces cerevisiae* for the endoplasmic reticulum-associated degradation of a multispanning membrane protein and a soluble luminal protein. J Biol Chem. 2004;279:38369–78. 10.1074/jbc.M402468200 15252059

[83] Ravid T Kreft SG Hochstrasser M. Membrane and soluble substrates of the Doa10 ubiquitin ligase are degraded by distinct pathways. EMBO J. 2006;25:533–43. 10.1038/sj.emboj.7600946 16437165 PMC1383530

[84] Knop M Finger A Braun T Hellmuth K Wolf DH. Der1, a novel protein specifically required for endoplasmic reticulum degradation in yeast. EMBO J. 1996;15:753–63. 10.1002/j.1460-2075.1996.tb00411.x 8631297 PMC450274

[85] Carvalho P Goder V Rapoport TA. Distinct ubiquitin ligase complexes define convergent pathways for the degradation of ER proteins. Cell. 2006;126:361–73. 10.1016/j.cell.2006.05.043 16873066

[86] Carroll SM Hampton RY. Usa1p is required for optimal function and regulation of the Hrd1p ER-associated degradation (ERAD) ubiquitin ligase. J Biol Chem. 2010;285:5146–56. 10.1074/jbc.M109.067876 19940128 PMC2820741

[87] Sato BK Schulz D Do PH Hampton RY. Misfolded membrane proteins are specifically recognized by the transmembrane domain of the Hrd1p ubiquitin ligase. Mol Cell. 2009;34:212–22. 10.1016/j.molcel.2009.03.010 19394298 PMC2710143

[88] Kanehara K Xie W Ng DT. Modularity of the Hrd1 ERAD complex underlies its diverse client range. J Cell Biol. 2010;188(5):707–16. 10.1083/jcb.200907055 20212318 PMC2835937

[89] Vashist S Ng DT. Misfolded proteins are sorted by a sequential checkpoint mechanism of ER quality control. J Cell Biol. 2004;165:41–52. 10.1083/jcb.200309132 15078901 PMC2172089

[90] Nakatsukasa K Huyer G Michaelis S Brodsky JL. Dissecting the ER-associated degradation of a misfolded polytopic membrane protein. Cell. 2008;132:101–12. 10.1016/j.cell.2007.11.023 18191224 PMC2219389

[91] Rabinovich E Kerem A Frohlich KU Diamant N Bar Nun S. AAA-ATPase p97/Cdc48p, a cytosolic chaperone required for endoplasmic reticulum-associated protein degradation. Mol Cell Biol. 2002;22:626–34. 10.1128/MCB.22.2.626-634.2002 11756557 PMC139744

[92] Ye Y Shibata Y Yun C Ron D Rapoport TA. A membrane protein complex mediates retro-translocation from the ER lumen into the cytosol. Nature. 2004;429:841–7. 10.1038/nature02656 15215856

[93] Carvalho P Stanley AM Rapoport TA. Retrotranslocation of a misfolded luminal ER protein by the ubiquitin-ligase Hrd1p. Cell. 2010;143(4):579–91. 10.1016/j.cell.2010.10.028 21074049 PMC3026631

[94] Suzuki T Park H Hollingsworth NM Sternglanz R Lennarz WJ. PNG1, a yeast gene encoding a highly conserved peptide: N-glycanase. J Cell Biol. 2000;149:1039–52. 10.1083/jcb.149.5.1039 10831608 PMC2174826

[95] Schauber C Chen L Tongaonkar P Vega I Lambertson D Potts W Rad23 links DNA repair to the ubiquitin/proteasome pathway. Nature. 1998;391:715–8. 10.1038/35661 9490418

[96] Kim I Mi K Rao H. Multiple interactions of rad23 suggest a mechanism for ubiquitylated substrate delivery important in proteolysis. Mol Biol Cell. 2004;15:3357–65. 10.1091/mbc.e03-11-0835 15121879 PMC452589

[97] Baek GH Kim I Rao H. TheCdc48 ATPase modulates the interaction between two proteolytic factors Ufd2 and Rad23. Proc Natl Acad Sci USA. 2011;108:13558–63. 10.1073/pnas.1104051108 21807993 PMC3158229

[98] Koskela EV de Ruijter JC Frey AD. Following nature’s roadmap: folding factors from plasma cells led to improvements in antibody secretion in *S. cerevisiae.* Biotechnol J. 2017;12:1–13. 10.1002/biot.201600631 28429845

[99] Liu J Han Q Cheng Q Chen Y Wang R Li X Efficient expression of human lysozyme through the increased gene dosage and co-expression of transcription factor Hac1p in *Pichia pastoris.* Curr Microbiol. 2020;77:846–54. 10.1007/s00284-019-01872-9 31932996

[100] Sheng J Flick H Feng X. Systematic optimization of protein secretory pathways in *Saccharomyces cerevisiae* to increase expression of hepatitis B small antigen. Front Microbiol. 2017;8:875–84. 10.3389/fmicb.2017.00875 28559891 PMC5432677

[101] Sallada ND Harkins LE Berger BW. Effect of gene copy number and chaperone coexpression on recombinant hydrophobin HFBI biosurfactant production in *Pichia pastoris.* Biotechnol Bioeng. 2019;116:2029–40. 10.1002/bit.26982 30934110

[102] Han M Wang W Zhou J Gong X Xu C Li Y, Li Q Activation of the unfolded protein response *via* co-expression of the HAC1i gene enhances expression of recombinant elastase in *Pichia pastoris.* Biotechnol Bioprocess Eng. 2020;25:302–7. 10.1007/s12257-019-0381-2

[103] Valkonen M Penttila M Saloheimo M. Effects of inactivation and constitutive expression of the unfolded protein response pathway on protein production in the yeast *Saccharomyces cerevisiae.* Appl Environ Microbiol. 2003;69:2065–72. 10.1128/AEM.69.4.2065-2072.2003 12676684 PMC154816

[104] Harmsen MM Bruyne MI Raue HA Maat J. Overexpression of binding protein and disruption of the *PMR1* gene synergistically stimulate secretion of bovine prochymosin but not plant thaumatin in yeast. Appl Microbiol Biotechnol. 1996;46:365–70. 10.1007/BF00166231 8987725

[105] de Ruijter JC Frey AD. Analysis of antibody production in *Saccharomyces cerevisiae*: effects of ER protein quality control disruption. Appl Microbiol Biotechnol. 2015;99:9061–71. 10.1007/s00253-015-6807-7 26184977

[106] Robinson AS Hines V Wittrup KD. Protein disulfide isomerase overexpression increases secretion of foreign proteins in *Saccharomyces cerevisiae.* Nat Biotechnol. 1994;12:381–4. 10.1038/nbt0494-381 7764684

[107] Inan M Aryasomayajula D Sinha J Meagher MM. Enhancement of protein secretion in *Pichia pastoris* by overexpression of protein disulfide isomerase. Biotechnol Bioeng. 2006;93:771–8. 10.1002/bit.20762 16255058

[108] Bartkevičiūtė D Sasnauskas K. Disruption of the *MNN10* gene enhances protein secretion in *Kluyveromyces lactis* and *Saccharomyces cerevisiae.* FEMS Yeast Res. 2004;4:833–40. 10.1016/j.femsyr.2004.03.001 15450190

[109] Friedlander R Jarosch E Urban J Volkwein C Sommer T. A regulatory link between ER-associated protein degradation and the unfolded-protein response. Nat Cell Biol. 2000;2:379–84. 10.1038/35017001 10878801

[110] Benitez EM Stolz A Wolf DH. Yos9, a control protein for misfolded glycosylated and non-glycosylated proteins in ERAD. FEBS Lett. 2011;585:3015–9. 10.1016/j.febslet.2011.08.021 21871892

[111] Izawa T Nagai H Endo T Nishikawa S. Yos9p and Hrd1p mediate ER retention of misfolded proteins for ER-associated degradation. Mol Biol Cell. 2012;23:1283–93. 10.1091/mbc.e11-08-0722 22298424 PMC3315801

[112] Raschmanová H Weninger A Knejzlík Z Melzoch K Kovar K. Engineering of the unfolded protein response pathway in *Pichia pastoris*: enhancing production of secreted recombinant proteins. Appl Microbiol Biotechnol. 2021;105:4397–414. 10.1007/s00253-021-11336-5 34037840 PMC8195892

[113] Tang H Song M He Y Wang J Wang S Shen Y Engineering vesicle trafficking improves the extracellular activity and surface display efficiency of cellulases in *Saccharomyces cerevisiae.* Biotechnol Biofuels. 2017;10:53. 10.1186/s13068-017-0738-8 28261326 PMC5327580

[114] Bao J Huang M Petranovic D Nielsen J. Moderate expression of *SEC16* increases protein secretion by *Saccharomyces cerevisiae.* Appl Environ Microbiol. 2017;83:e03400–16. 10.1128/AEM.03400-16 28476767 PMC5494634

[115] Huang M Bai Y Sjostrom SL Hallström BM Liu Z Petranovic D Microfluidic screening and whole-genome sequencing identifies mutations associated with improved protein secretion by yeast. Proc Natl Acad Sci USA. 2015;112:E4689–96. 10.1073/pnas.1506460112 26261321 PMC4553813

[116] Shusta EV Raines RT Pluckthun A Wittrup KD. Increasing the secretory capacity of *Saccharomyces cerevisiae* for production of single-chain antibody fragments. Nat Biotechnol. 1998;16:773–7. 10.1038/nbt0898-773 9702778

[117] Suzuki H Imaeda T Kitagawa T Kohda K. Deglycosylation of cellulosomal enzyme enhances cellulosome assembly in *Saccharomyces cerevisiae.* J Biotechnol. 2012;157:64–70. 10.1016/j.jbiotec.2011.11.015 22154562

[118] Nagasu T Shimma Y Nakanishi Y Kuromitsu J Iwama K Nakayama K Isolation of new temperature-sensitive mutants of *Saccharomyces cerevisiae* deficient in mannose outer chain elongation. Yeast. 1992;8:535–47. 10.1002/yea.320080705 1523886

[119] Wang TY Huang CJ Chen HL Ho PC Ke HM Cho HY Systematic screening of glycosylation- and trafficking-associated gene knockouts in *Saccharomyces cerevisiae* identifies mutants with improved heterologous exocellulase activity and host secretion. BMC Biotechnol. 2013;13:71. 10.1186/1472-6750-13-71 24004614 PMC3766678

[120] Yu Y Liu Z Chen M Yang M Li L Mou H. Enhancing the expression of recombinant κ-carrageenase in *Pichia pastoris* using dual promoters, co-expressing chaperones and transcription factors. Biocatal Biotransform. 2020;38:1–10. 10.1080/10242422.2019.1655001

[121] Ben Azoun S Belhaj AE Göngrich R Gasser B Kallel H. Molecular optimization of rabies virus glycoprotein expression in *Pichia pastoris.* Microb Biotechnol. 2016;9:355–68. 10.1111/1751-7915.12350 26880068 PMC4835572

[122] Huang M Wang G Qin J Petranovic D Nielsen J. Engineering the protein secretory pathway of *Saccharomyces cerevisiae* enables improved protein production. Proc Natl Acad Sci USA. 2018;115:E11025–32. 10.1073/pnas.1809921115 30397111 PMC6255153

[123] Besada-Lombana PB Da Silva NA. Engineering the early secretory pathway for increased protein secretion in *Saccharomyces cerevisiae.* Metab Eng. 2019;55:142–51. 10.1016/j.ymben.2019.06.010 31220665

